# Morphoea with Myositis: A Rare Association

**DOI:** 10.1155/2011/134705

**Published:** 2011-10-20

**Authors:** Mary Sommerlad, Richard Bull, Claire Gorman

**Affiliations:** ^1^Department of Rheumatology, Homerton University Hospital, London E9 6SR, UK; ^2^Department of Dermatology, Homerton University Hospital, London E9 6SR, UK

## Abstract

In this case, we describe an unusual presentation of a young woman with a rash typical of morphoea (confirmed on biopsy), who went on to develop myositis in an atypical distribution. Although the association of myositis with diffuse systemic sclerosis is well described, the link with localised scleroderma (morphoea) and myositis has not been described.

## 1. Case History

**Figure 1:**
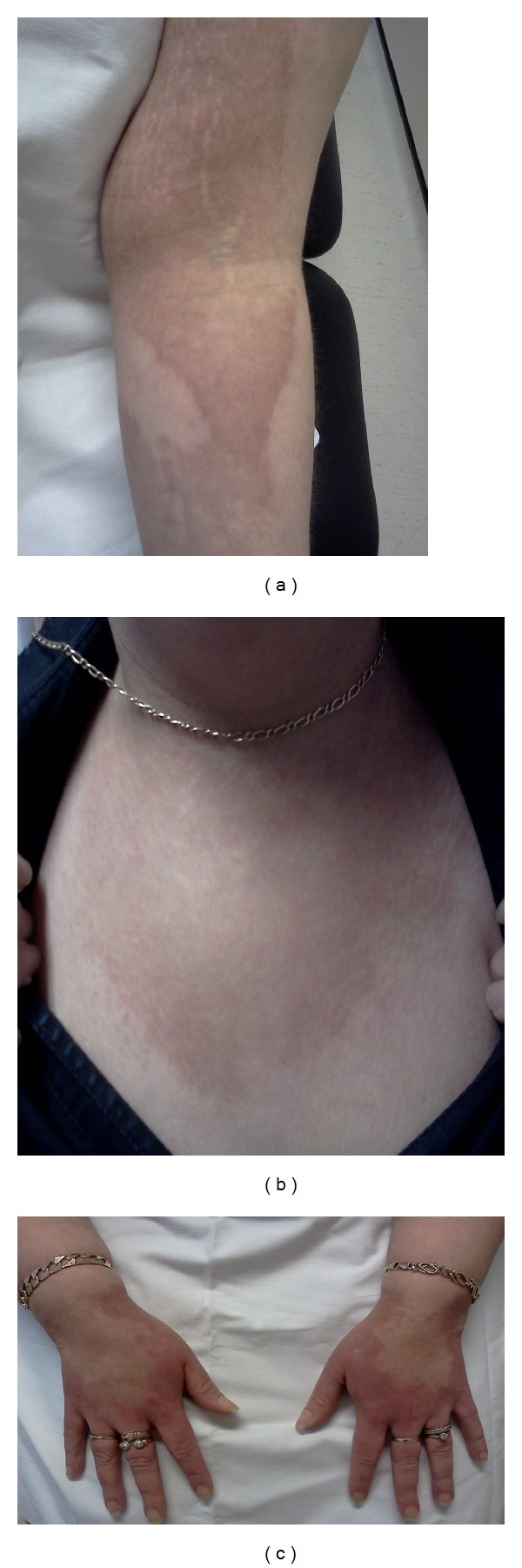
Atrophic eruption affecting (a) flexures of arms, (b) upper chest, and (c) hands.

A 33 year old previously fit and well Caucasian woman presented to the Dermatology clinic with a tender atrophic eruption involving the flexures of both arms, dorsum of hands, chest and back (see Figure 1) associated with nail changes. A skin biopsy at the time showed broad bands of sclerotic hyalinised collagen in the dermis with loss of elastic tissue and a mild perivascular lymphocytic infiltrate. As a connective tissue disorder was suspected, she underwent nailfold capillaroscopy which revealed a grossly abnormal pattern of capillary network consistent with an autoimmune connective tissue disease.

She was diagnosed with *Atrophoderma of Pasini and Pierini, *a form of dermal atrophy of unknown aetiology which can be associated with the spirochete *Borrelia burgdorferi,* and is transmitted by tick bites. Half of patients with *Atrophoderma of Pasini and Pierini *having positive *B. burgdorferi* serology [1]. Although *Borrelia burgdorferi* serology was negative and there was no recollection of any tick bites, she received a course of oral Doxycyline. This did not improve her skin condition and, as the eruption evolved, she was subsequently diagnosed with *morphoea* due to the clinical appearance of her skin lesions. She was duly started on topical steroids with a view to having a course of phototherapy.

Five months later, she developed weakness and pain in both her upper arms, describing difficulty carrying shopping bags and combing her hair. She was referred to the Rheumatology department for further assessment; plans for phototherapy were abandoned. On examination she had weakness in the proximal muscles of both upper limbs with no wasting. She did not have any other symptoms consistent with systemic sclerosis or any other connective tissue disease. Her chest was clear to auscultation, and heart sounds were normal. Urinalysis was unremarkable. 

Autoantibody profile was negative (ANA, ENA, and Rheumatoid factor), and ESR and CRP were within the normal range. CK was moderately raised at 490 iu/L (normal range 25–190 iu/L) as was LDH at 264 iu/L (normal 70–250 iu/L) although AST was within the normal range.

**Figure 2:**
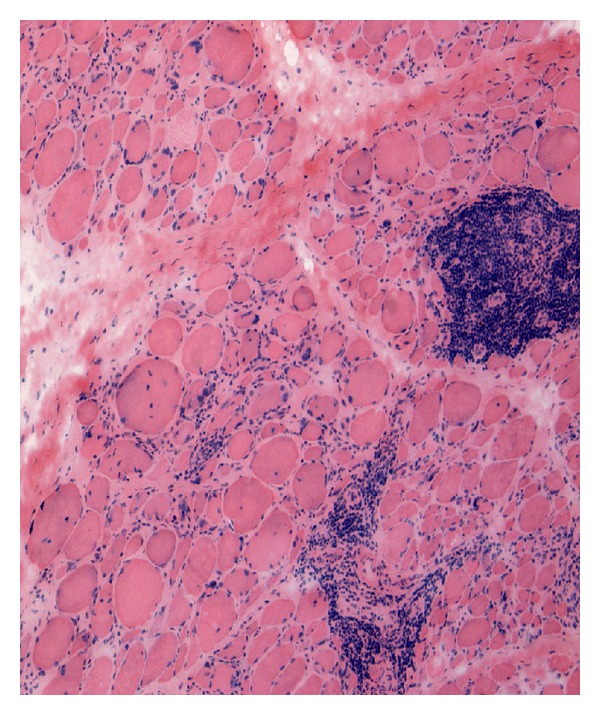
Muscle biopsy demonstrating gross lymphocyte infiltration.

**Figure 3:**
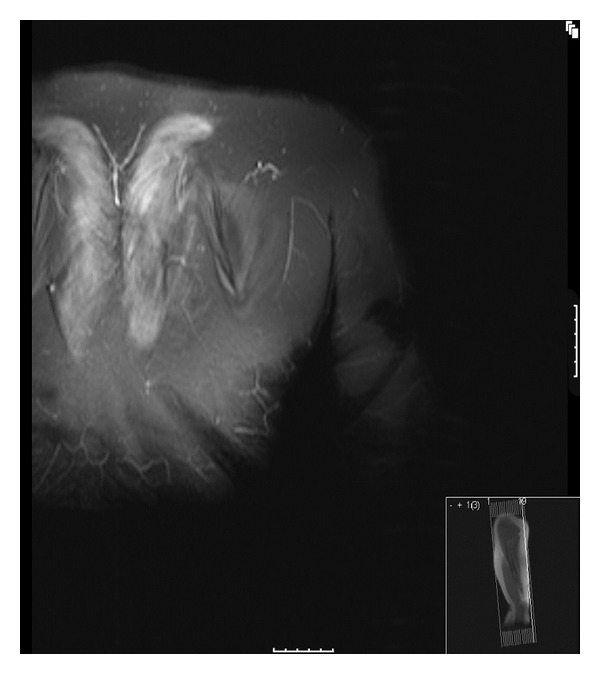
Bilateral increased intensity of the trapezius in the STIR sequence (MRI sagittal section in smaller box).

MRI of the upper limbs demonstrated increased intensity in the trapezius muscle bilaterally (see Figure 2). Muscle biopsy demonstrated a profuse lymphocytic infiltration—immunostaining excluded a lymphomatous process. Because the infiltrate was predominantly perivascular and there were some follicle-like structures along with perifascular atrophy, the possibility of dermatomyositis was raised. However, generally, the features were thought to be consistent with an inflammatory myopathy (see Figure 3). She was commenced on Methotrexate and Prednisolone bridging therapy.

CT scans of the chest, abdomen, and pelvis did not reveal any neoplastic lesion, and a range of tumour markers were also negative (CA125, CA153, and CA199). Her muscular symptoms improved with immunosuppression, and her CK fell from 490 iu/L to 275 iu/L. However, her skin disease remains resistant to immunosuppressive therapy, and she has started a course of phototherapy. Her working diagnosis at present is *morphoea with myositis.*

## 2. Discussion

Morphoea with myositis is not well described in the literature. A PubMED literature search retrieved 122 articles for morphoea and myositis. The majority of these articles describe an association between systemic sclerosis and myositis usually in a setting of established scleroderma disease [2]. The most similar case identified was described by Warin in 1973 and described a young man with morphoea overlying myositis; unlike our case, this patient did have a raised ESR and was ANA positive (ENA was not assessed [3]). 

The term *morphoea profunda *has been coined to describe inflammation extending from the dermis to the superficial muscle layers with or without the production of autoantibodies [4]. Melani et al. described a 40-year-old patient who initially presented with morphoea profunda (with normal ESR and CRP and a negative autoantibody profile) which evolved into systemic sclerosis over several years [5]. The unusual elements of this case are that the patient has not developed any autoantibodies and that her inflammatory markers have remained within normal limits, that is, the scleroderma remains localised. Furthermore, her dermis is relatively spared while her muscle demonstrates gross infiltration with lymphocytes. Although the cause of her disease is unknown, it remains important to follow up this patient in order to identify any evolution of the disease.
